# Contemporary Approaches to Pain Assessment and Management: A Clinical Perspective

**DOI:** 10.7759/cureus.109593

**Published:** 2026-05-25

**Authors:** Maheshvari N Patel, Nayan Patel

**Affiliations:** 1 Clinical Research, NovoBliss Research Private Limited, Ahmedabad, IND; 2 Pharmacology, Swaminarayan University, Ahmedabad, IND; 3 Clinical Research Operations, NovoBliss Research Private Limited, Ahmedabad, IND

**Keywords:** chronic pain, neuroimaging, pain measurement, patient-reported outcome measures, quantitative sensory testing

## Abstract

Chronic pain is a complex phenomenon whose assessment involves not only physiological but also psychological and social components. Due to its subjective and variable nature, accurately measuring chronic pain remains challenging in both clinical practice and research settings. Various assessment approaches have been developed to evaluate pain and its impact on patients; however, many commonly used methods, particularly unidimensional tools, primarily focus on pain intensity and often fail to capture its broader effects on affective, cognitive, and functional domains.

Recent advances in pain science have led to the development of more objective and mechanistically informative evaluation techniques. Approaches that assess sensory function provide insights into peripheral and central pain processing, while advanced imaging technologies have improved understanding of brain mechanisms and neural adaptations associated with chronic pain. In addition, emerging sensor-based devices and digital health tools enable continuous, real-time monitoring of physiological and functional parameters related to pain. These developments align with evolving conceptual frameworks that recognize chronic pain as a distinct clinical condition rather than merely a symptom.

The integration of conventional assessment approaches with advanced multidimensional techniques holds significant potential to enhance the accuracy and clinical relevance of pain evaluation. This scientific communication aims to highlight current methodologies, identify existing challenges, and explore future directions, emphasizing the importance of a holistic and standardized approach to pain assessment.

## Editorial

Pain is a complex and multifaceted experience that continues to be among the most common grounds for seeking medical help in the world. Pain is increasingly recognized as more than just a symptom; rather, it can be seen as a pathological entity that has a considerable influence on psychological well-being, physical functioning, and general quality of life. According to the International Association for the Study of Pain, pain is defined as “an unpleasant sensory and emotional experience associated with or similar to actual or potential tissue damage” [1]. Specifically, chronic pain constitutes a considerable global public health issue due to the associated disability and low productivity level [2].

Despite all developments that have occurred in the understanding of pain mechanisms, the evaluation of pain remains largely centered on commonly used rating scales such as the Numeric Rating Scale (NRS), Visual Analog Scale (VAS), and Verbal Rating Scale (VRS). Although these measurement tools are straightforward and widely adopted, they primarily assess pain intensity and often do not fully capture the qualitative, affective, and functional dimensions of pain. In condition-specific contexts, instruments such as the Western Ontario and McMaster Universities Osteoarthritis Index (WOMAC) have been developed to assess pain alongside physical function and stiffness, particularly in patients with osteoarthritis (OA). While WOMAC provides a more comprehensive and function-oriented evaluation, it is limited to specific clinical populations with hip and knee OA, especially elderly people, and may not be broadly applicable across diverse pain conditions, because it is specially developed to assess the three major problems associated with hip and knee OA [3]. Furthermore, the subjective nature of these measurement scales makes them susceptible to variability influenced by an individual's psychological and physiological state [4,5].

During years of development, a shift in paradigm has occurred toward a more pragmatic view of pain, which is propelled by progressions in psychophysics, neurobiology, and digital health sciences. Future methodologies like quantified sensory examination, biomarker discovery, neuroimaging procedures, and patient-based outcome measures have broadened the scope of pain assessment beyond intensity measurement. These methodologies intend to offer semi-objective and objective perspectives on mechanisms involved and further elaborate on the characteristics of pain disorders [6,7].

With an increasing appreciation for the multifaceted nature of pain, there have been attempts to integrate the evaluation process by including subjective reporting along with physiological and physical measurements. Such evaluations take into consideration not only the physiological aspects but also the functional, behavioral, and psychological factors that affect the experience of pain. Among these, the physical aspect of pain refers to the effect of pain on movement, functioning, sleep, and other activities.

It is obvious that pain may have a serious negative impact on physical functioning, decrease involvement in usual activities, and cause fatigue. The measurement of functional limitations is critical for obtaining the necessary information about the real impact of pain and consequences for patients’ health and quality of life. Several scales and objective measurements are commonly used to evaluate physical limitations associated with pain. In addition, objective performance-based assessments, including the Timed Up and Go Test and the Six-Minute Walk Test, provide important measures of mobility, gait performance, endurance, and functional independence. These assessments are clinically significant, as greater pain severity is often correlated with poorer physical performance and increased functional disability [8].

This approach is consistent with the biopsychosocial model of pain, according to which various factors such as depression and anxiety play a role in the development of pain perception and significantly influence chronic pain perception and physical functioning, which can be assessed using validated instruments like the Hospital Anxiety and Depression Scale (HADS), Beck Depression Inventory, and Patient Health Questionnaire-9 [9].

In this context, the current scientific publication is intended to offer a brief summary of the methods for assessing pain in the modern world, emphasizing the shift from classical subjective measurement scales to modern multidimensional and technology-based approaches. In this respect, this study intends to add to the existing discussion on the need for an improved approach to the assessment of pain.

Conceptual framework of pain

Pain is considered a multifaceted phenomenon that includes sensory, emotional, cognitive, and social dimensions. The modern conception of pain cannot be confined within the framework of the biomedical paradigm since pain is associated with the interaction between physical processes and psychological and environmental factors. Indeed, this shift in the conception of pain can be illustrated by the new definition “An unpleasant sensory and emotional experience associated with actual or potential tissue damage, or described in terms of such damage” provided by the International Association for the Study of Pain. This definition emphasizes that pain not only is the outcome of tissue damage but also involves the emotional and psychological elements [1].

In terms of its mechanism, pain can be divided into three main categories: nociceptive, neuropathic, and nociplastic pain. Nociceptive pain is caused by any actual or potential injury to non-neural tissues. Neuropathic pain, on the other hand, occurs when there is a lesion or disease that has affected the somatosensory nervous system; symptoms include burning, tingling, and electric shock-like pain [10]. A new concept in pain mechanism is nociplastic pain, which refers to pain that is caused by abnormal nociception even if there is no sign of injury to the tissues or a lesion to the somatosensory system, as found in fibromyalgia patients [11].

Pain can also be divided into two major types on the basis of duration, namely, acute and chronic. Acute pain often acts as a defense mechanism, which helps us to detect tissue damage and heal from it. Chronic pain, which refers to the pain that persists for more than the expected time required for healing of tissues (usually greater than three months), is considered a different medical entity rather than just being a sign of the disease [12].

The biopsychosocial model offers a framework that helps understand pain by taking into account biological processes in relation to mental states, such as mood, cognition, and coping as well as social context. It emphasizes the idea that pain is not only driven by sensory nociception but also influenced by personal experience and expectation. Therefore, an appropriate assessment of pain should take into consideration all of these factors and not be based on just one of them.

Generally speaking, there has been a shift in the way of understanding pain toward a more holistic approach. The understanding of the complexity of pain mechanisms and experiences is crucial to pain assessment in today's medicine.

Conventional pain assessment tools

Various tools are available to evaluate pain in patients, each with specific advantages and limitations. Commonly used unidimensional tools such as the VAS and NRS are simple, rapid, and easy to administer for assessing pain intensity, with the NRS frequently recommended in both clinical practice and research because of its convenience for verbal and written administration. These scales are clinically significant for monitoring symptom severity, evaluating treatment response, and facilitating communication between patients and healthcare professionals. However, both primarily assess pain intensity and do not provide a multidimensional evaluation of the pain experience. Therefore, more comprehensive assessment tools have been developed. The McGill Pain Questionnaire (MPQ) evaluates both the intensity and qualitative characteristics of pain, allowing assessment of sensory and affective dimensions. Similarly, the Chronic Pain Grade Scale measures chronic pain severity and its impact on daily functioning, while the Short Form-36 Bodily Pain Scale assesses pain in relation to overall health and quality of life, enabling comparisons across different populations and clinical conditions [5]. Such tools provide a more comprehensive and functional assessment within specific clinical contexts while complementing traditional intensity-based measures.

The VAS usually comprises a 10 cm scale labeled with descriptions like "no pain" and "worst possible pain," giving patients the option to point out their degree of pain sensation. On the other hand, the NRS is based on a numeric scale, mostly ranging between 0 and 10, whereas the VRS classifies pain on the basis of descriptions like mild, moderate, and severe [11]. These methods have been proven reliable and responsive in acute pain assessments.

Even though these traditional methods are commonly employed, they have multiple drawbacks that must be taken into account. For one thing, they are based on self-reports, which can vary according to personal pain tolerance levels, emotions, culture, and mental processes. This means that there may be large differences between individuals that will inevitably influence the results [13]. Furthermore, these pain measurement methods focus on pain intensity but fail to measure other vital parameters, including pain characteristics, duration, functionality, and psychological effects.

Whereas standard pain assessments may prove very useful in many cases, they can also be further improved in more complicated chronic pain cases, for instance, through the use of other types of assessments that are complementary to them. Chronic pain can, indeed, prove dynamic and be affected by processes like central sensitization, and other modes of assessing this type of pain can be useful. Another example of when special consideration should be taken is during the assessment of pain in special populations.

In order to overcome these deficiencies, multidimensional scales like the MPQ and the Brief Pain Inventory (BPI) have been devised. The MPQ and BPI include a series of well-structured queries and descriptions that are intended to be easily comprehensible by patients. Through the inclusion of sensory, emotional, and functional aspects, they offer a more complete assessment of pain experience [14,15]. However, their increased complexity and time requirements may limit routine clinical use. Generally, despite the importance of traditional methods used to measure pain, it is evident that the weaknesses associated with such methods underscore the need to adopt other pain measurement methods that can be more effective in addressing the multifaceted aspects of pain.

Emerging and advanced pain evaluation techniques

As a result of advances in research on and technology related to pain, there have been many innovative assessment tools created that go beyond the conventional subjective measures of pain. Such new tools seek to bring forth an objective or multidimensional perspective of the processes behind pain in order to increase diagnostic precision and stratification.

Quantitative Sensory Testing

Quantitative sensory testing (QST) is a psychophysical technique employed to determine the functional status of sensory nerves based on their response to controlled sensory stimuli such as temperature, pressure, and vibration. QST tests enable the examination of sensory pathways from peripheral to central nervous system levels and have proven useful in studying neuropathic pain states. The standardized QST protocol is designed to detect sensory dysfunctions like hyperalgesia and allodynia [16]. Despite its utility, QST requires specialized equipment and trained personnel, and its results may still be influenced by patient cooperation and cognitive factors, limiting its routine clinical applicability.

Neuroimaging Techniques

The use of neuroimaging has been pivotal in furthering knowledge of pain mechanisms, particularly in observing the areas of the brain that are part of the process, which have been labeled as the “pain matrix.” Through functional MRI (fMRI) and PET, one can study the brain activity related to the nociceptive and emotional aspects of pain [17].

Neuroimaging research employing fMRI, resting-state fMRI, and PET demonstrates structural and functional alterations in pain processing domains, including the anterior cingulate cortex, insula, thalamus, and prefrontal cortex, among individuals with chronic pain disorders such as fibromyalgia and chronic low back pain. Some studies also find that when the subjects are compared with healthy humans, they showed regional gray matter alterations, functional connectivity reduction within descending pain-modulatory pathways, and enhanced activation of the pain matrix, which supports the occurrence of altered central pain processing and sensitization mechanisms [18]. Thus, the findings advanced the knowledge of the physiology of chronic pain and might help in the emergence of imaging biomarkers for diagnosis, symptom monitoring, and assessment of treatment response. Nevertheless, neuroimaging's usefulness for pain evaluation purposes is somewhat constrained by its high cost, technical difficulty, and difficulty interpreting results on an individual basis. At present, these methods are used mainly for research purposes and not for clinical evaluations.

Biomarkers in Pain Evaluation

The search for biological markers for pain has proven to be an interesting field of research that could bring objectivity to pain assessment. Biological markers, such as pro-inflammatory cytokines (interleukin-6, tumor necrosis factor-alpha), neuropeptides, and polymorphisms of genes, have been studied due to their possible involvement in the modulation of pain or the vulnerability to chronic pain [19].

Even though these markers provide information on the pathophysiology involved in a certain painful condition, their application in a clinical setting is difficult due to problems with sensitivity, variability, and thresholds. Therefore, none of these markers is currently used in practice.

Digital and Wearable Technologies

The incorporation of digital health tools in pain assessment techniques has opened up new avenues for continuous and real-time monitoring of the condition. Using mobile apps and ePain diaries can help individuals record their pain levels, triggering factors, and functioning capabilities, thereby minimizing the recall bias and providing accurate information [20]. Additionally, wearable devices have made pain assessments even more effective by measuring physiological variables like physical activity, sleep patterns, and heart rate variability (HRV), which may be linked to pain sensations. These technologies support longitudinal tracking and personalized pain profiling; however, challenges related to data privacy, user adherence, and standardization remain to be addressed.

**Figure 1:**
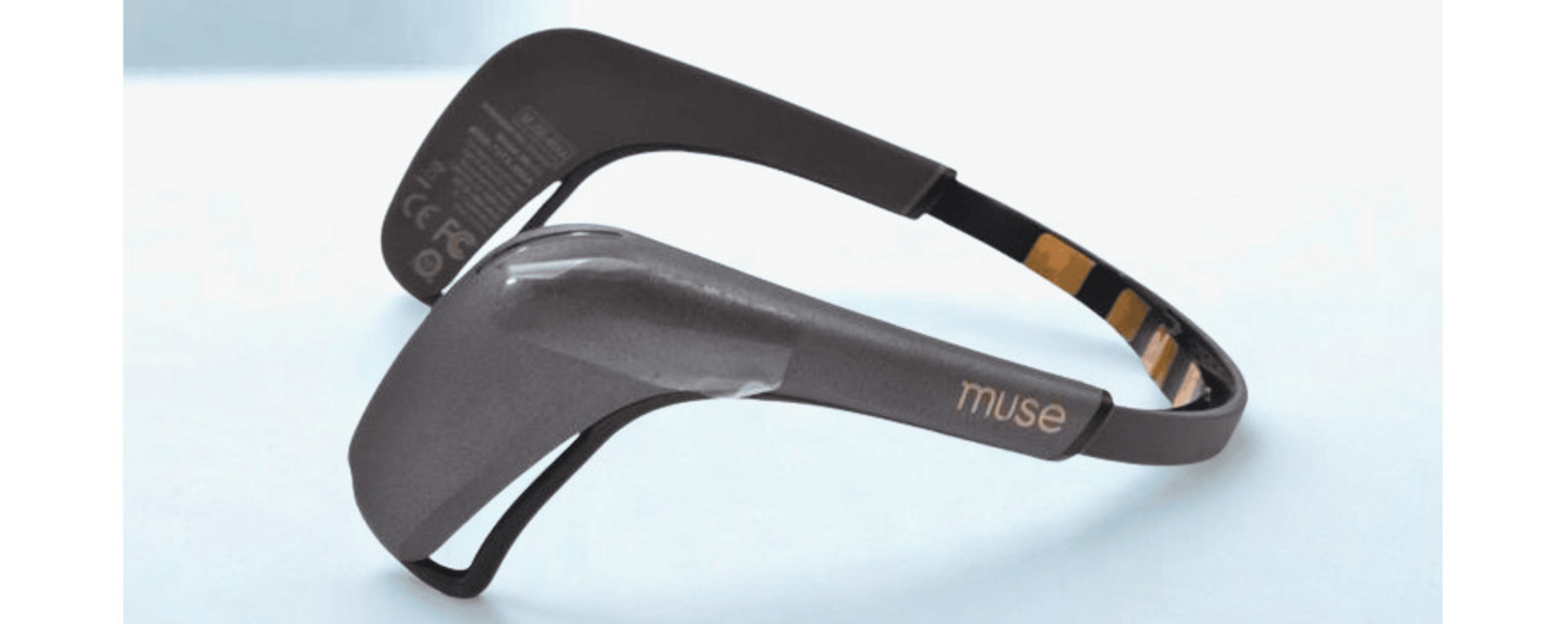
Muse headband Digital and wearable technology for the assessment of pain. Image captured from the actual instrument available at the authors' facility.

Emerging biosensor wearables like electroencephalography (EEG)-based headbands, like the Muse headband (InteraXon, Toronto, Canada), have allowed for the detection of brain activity in a non-invasive manner (Figure 1). Neural activity that correlates with cognition and affect can be captured using such devices. The ability of such a wearable to detect neural oscillations that correlate with pain perception makes them a promising method for pain assessment [21]. However, more validation is needed to confirm the utility of the devices in pain evaluation.

Patient-Reported Outcome Measures

Patient-reported outcome measures (PROMs) are considered a multidimensional assessment approach that includes not only the measurement of the subjective experience of pain but also its consequences related to functional status and quality of life. The application of PROMs is quite widespread in clinical practice as well as scientific research; nevertheless, the nature of self-representation in these measures and the risk of respondent burden can make them less valuable for certain patients.

In general, all of these techniques indicate a tendency toward multidimensional and mechanisms-oriented evaluation of pain. Although all methods have their advantages and disadvantages, their combination may be beneficial in assessing pain in modern medicine.

Integration of multidimensional pain assessment

As research into pain has evolved and recognized that pain is a complex and multidimensional phenomenon, a multifaceted method for evaluating it is required. Since no single measurement instrument can measure the intricacies of pain, a modern method involves the utilization of complementary techniques. An example of such a strategy would be the biopsychosocial perspective on pain, which recognizes that pain is influenced by the interactions between biological, psychological, and sociological factors. For clinical purposes, multiple approaches may be utilized simultaneously to assess pain by using patient-reported outcomes such as pain intensity rating scales and PROMs, along with the utilization of objective or semiobjective assessment methods like QST or functional testing. Self-reporting provides information about the subjective experience of the pain suffered by the individual, while objective measures can help in understanding the possible mechanisms that might be contributing to the pain condition, such as peripheral sensitization or central nervous system involvement [22]. The combination of all these tools allows a better phenotyping of pain, which is critical in comprehending the variation of pain presentation between different patients.

Additionally, the inclusion of functional outcomes, such as activity, sleep, and performance, increases the clinical significance of pain assessments because these symptoms have been associated with pain intensity and disability [23]. This advancement is possible thanks to digital health technologies that monitor patient self-reports and physiological data in real-time through wearable devices.

Moreover, multidimensional assessment models may contribute to moving pain management into the realm of precision medicine. Integration of various forms of information may enable more accurate patient stratification according to their specific pathophysiology and symptomatology [24]. While implementing multidimensional models for pain assessment on a routine basis poses difficulties because of standardization problems and associated expenses, their benefits cannot be overlooked.

In summary, the use of multidimensional instruments in pain assessment marks a milestone in the field of pain management. It shifts the focus from singular evaluation to a more holistic model of assessment that can aid in developing a treatment plan that is more precise and efficient.

Challenges and future directions

Despite the marked improvements in the methods used to assess pain, a number of obstacles prevent its more comprehensive application. One of the key problems lies in the absence of standardization in testing procedures. It makes the use of advanced testing methods more complicated due to their difficulty in comparing to other findings and incorporating into the usual practice of clinicians.

Another crucial constraint involves the availability and affordability of novel technology. Methods like neuroimaging and QST involve the need for specific instruments and highly trained personnel who can interpret the results, and the conditions under which they operate are not always found in all healthcare facilities, especially in developing countries. The analysis of the results obtained through these methods is also complicated.

The search for accurate and validated biomarkers of pain remains ongoing. Despite extensive research into biological markers such as inflammatory cytokines and neurochemical mediators, no biomarker has yet demonstrated sufficient reliability and specificity to be established as a definitive diagnostic tool. This highlights the persistent gap in objective pain assessment and underscores the need for more robust and reproducible indicators.

Ethical considerations also arise with the increasing use of advanced technologies, particularly neuroimaging and EEG-based approaches. Pain is closely linked to dynamic brain activity, involving complex interactions across multiple neural networks responsible for sensory, emotional, and cognitive processing. Techniques such as EEG and functional imaging attempt to capture these pain-brain interactions by identifying neural signatures associated with pain perception. However, interpreting such brain-derived data presents challenges related to individual variability, contextual influences, and the risk of overgeneralization. Moreover, reliance on brain-based metrics raises concerns regarding privacy, potential misuse, and the overemphasis on objective findings in a fundamentally subjective experience. It is therefore essential that these technologies are used to complement, rather than replace, patient-reported outcomes, ensuring that the individual’s lived experience of pain remains central to evaluation.

In terms of the future, integrating artificial intelligence and machine learning can prove to be a very promising route to take. Both of these technologies make it possible to analyze large amounts of data collected from various sources. In terms of pain, clinical, behavioral, and physiologic information can be collected and analyzed.

The future work in this field must be directed at creating standardized, reliable, and easily available tools that could be seamlessly integrated into clinical practice. The necessity to develop the link between scientific achievements and their practical implementation in medicine deserves special attention. In general, solving these problems is crucial for moving forward to more objective and personalized pain assessment paradigms.

Conclusion

Pain assessment has evolved into a multidimensional process integrating advances in neurobiology, psychophysics, and digital health. While conventional assessment tools remain valuable for their simplicity and ease of use, they are limited in capturing the complexity of pain as a subjective experience. Condition-specific instruments further emphasize the importance of evaluating functional impairment alongside pain intensity. Emerging approaches, including brain activity-based techniques and advanced imaging methods, provide insights into pain-brain interactions and offer objective support to traditional self-reported measures. However, challenges related to standardization, accessibility, and clinical implementation persist.

Consequently, improving pain assessment requires an approach that integrates objective advancements with the subjective perception of the patient. Such a combination has the potential to enhance the reliability of evaluation and support more informed clinical decision-making, ultimately improving patient outcomes.
